# Supplementary material for: Diagnosis of Human Leptospirosis: Comparison of Microscopic Agglutination Test with Recombinant LigA/B Antigen-Based In-House IgM Dot ELISA Dipstick Test and Latex Agglutination Test Using Bayesian Latent Class Model and MAT as Gold Standard
Source: Diagnostics (Basel). 2022 Jun 13;12(6):1455. doi: 10.3390/diagnostics12061455 (PMC9221883; doi:10.3390/diagnostics12061455)
Supplement: Supplementary file 1 [file diagnostics-12-01455-s001.zip › diagnostics-1716237-supplementary.pdf]

Supplementary Table S1A

MAT (Acute only)	In-house recombinant LigBCon1-5 antigen based IgM Dot ELISA Dipstick test	Number (n=340)
Positive	Positive	51
Positive	Negative	00
Negative	Positive	01
Negative	Negative	288

Supplementary Table S1B

Total MAT (Acute or paired)	In-house recombinant LigBCon1-5 antigen based IgM Dot ELISA Dipstick test	Number (n=340)
Positive	Positive	63
Positive	Negative	03
Negative	Positive	01
Negative	Negative	273

Supplementary Table S2A

MAT (Acute only)	In-house recombinant LigBCon1-5 antigen based Latex Agglutination Test	Number (n=340)
Positive	Positive	51
Positive	Negative	00
Negative	Positive	01
Negative	Negative	288

Supplementary Table S2B

Total MAT (Acute or paired)	In-house recombinant LigBCon1-5 antigen based Latex Agglutination Test	Number (n=340)
Positive	Positive	60
Positive	Negative	06
Negative	Positive	01
Negative	Negative	273
